# Using Waste Plastics as Asphalt Modifier: A Review

**DOI:** 10.3390/ma15010110

**Published:** 2021-12-24

**Authors:** Fengchi Xu, Yao Zhao, Kangjian Li

**Affiliations:** College of Civil Engineering, Nanjing Forestry University, Nanjing 210037, China; echo23336@163.com (F.X.); kangjian0119@163.com (K.L.)

**Keywords:** recycled waste plastic, asphalt binder and mixture, composite modification, performance, compatibility

## Abstract

The use of waste products in the production of asphalt binders and asphalt mixtures has become widespread due to economic and environmental benefits. In particular, the use of recycled waste plastic in asphalt binders and mixtures is gaining more attention. This review presents analyses and comparisons of various forms of waste plastic used in asphalt modification, and approaches to incorporating waste plastic into asphalt mixtures, both for single and composite modifications. It focuses on the properties of waste plastics, asphalt binders, and asphalt mixtures. Overall, the incorporation of plastic waste into asphalt mixtures can significantly improve high-temperature performance and has potential economic and environmental benefits. The performance of modified asphalt is highly dependent on multiple factors, such as waste sources, waste plastic dosages, blending conditions, and the pretreatment methods for waste plastic. There are different ways to apply waste plastics to blend into a mixture. In addition, this paper discusses the current challenges for waste plastic-modified asphalt, including the stability, low-temperature performance, modification mechanism, and laboratory problems of the blends. The use of chemical methods, such as additives and functionalization, is considered an effective way to achieve better interactions between waste plastics and the binder, as well as achieving a higher sufficiency utilization rate of waste plastics. Although both methods provide alternative options to produce waste plastic-modified asphalt with stability and high performance, the optimal proportion of materials used in the blends and the microcosmic mechanism of composite modified asphalt are not clear, and should be explored further.

## 1. Introduction

### 1.1. Environmental Problems Caused by Plastic Waste

**Figure 1:**
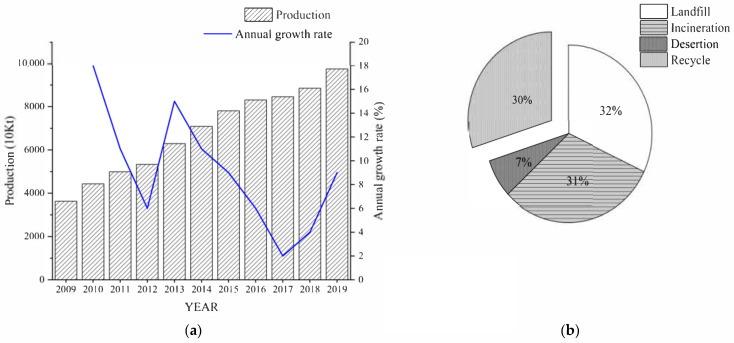
The production and disposal of plastics in China: (**a**) primary plastics production (2009–2019); (**b**) disposal of plastic waste in 2019.

Plastics have been widely used in diverse fields for their substantial benefits in terms of cost-effectiveness, light weight, durability, and ease of processing relative to many other materials [1,2]. The increasing demand in various fields promotes the rapid growth of the productivity of plastics. In 2019, the global production of plastics reached 368 million metric tons. China is one of the largest producers, accounting for around 30% [3]. China’s plastics industry is in a stage of rapid development in this context. For primary plastics production, it has increased by an average annual rate of 9.1% during the past 10 years, as shown in Figure 1a [4].

The widespread use of plastics brings great convenience to everyday life and promotes economic and social development. However, approximately 50% of plastic materials are single-use materials, such as packaging, agricultural films, and disposable consumer items. Between 20 and 25% of plastics are used for long-term infrastructures such as pipes, cable coatings, and structural materials [1]. At the same time, the huge amount of plastic consumption has led to stress on plastic waste management and eco-environmental protection. Currently, many countries have various management strategies for the total municipal solid waste (MSW) stream. Unfortunately, most of the world’s postconsumer plastic materials are treated as waste. Plastic waste in the US, China, and other countries has long been disposed of simply by landfill and incineration (for energy recovery). In 2018, landfills in the US received 27 million tons of plastic, accounting for 18.5% of all MSW landfilled [5]. Landfill, as a traditional plastic waste disposal method, has a high demand for land resources, which has also become a key issue for plastic waste disposal in many countries [2]. The long-term risk of chemicals leaching from the plastics into soils and waters is a serious environmental threat [6]. Moreover, the most severe issue is that this leaching of chemicals may take at least several decades, and probably centuries, as most plastics are not biodegradable. On the other hand, incineration effectively reduces both the volume and mass of plastic waste, but air pollution occurs during the heat treatment process in the incinerator, releasing carbon monoxide, dioxin, and other toxic emissions [7]. The waste plastics also contain heavy metals such as cadmium (Cd) and lead (Pb), which discharge from smoke dust and residues produced during the process of incineration [8].

Additionally, the improper handling of plastic waste leads to a high content of plastics in MSW incineration-bottom ash (MSWI-BA). The disposal of MSWI-BA results in an increase in contaminants that pollute water, soil, and oceans [9,10,11]. Microplastics derived from the fragmentation and degradation of plastics pose an even more serious concern for public health, as they are small enough to pass through waste filtration systems and are difficult to remove [12,13]. Consequently, “plastic pollution” or “white pollution” has become one of the most pressing environmental issues of the modern world [14].

The recycling and utilization of plastic waste have been considered a golden management strategy for reducing environmental impact and natural resource depletion [1,15]. With the increasing awareness of the dangers of improper plastic treatment, this issue has also attracted wide international attention. Countries around the world have introduced policies to ban the use of plastics. By 2020, most countries had begun to prohibit the use of disposable plastic products, as shown in Table 1. From 2008 to 2016, the consumption of plastic shopping bags in supermarkets decreased by more than 2/3, a cumulative reduction of about 1.4 million tons of plastic shopping bags, equivalent to a reduction of nearly 30 million tons of carbon dioxide in China. At the same time, some plastic waste enterprises quickly adjusted to establish and run a waste plastic industry recycling system to support the government strategy, which was shown to work effectively in China. In 2019, approximately 30% of plastic waste was recycled in China, as shown in Figure 1b [16].

### 1.2. The Benefits of Using Waste Plastic in Asphalt

China’s road network has developed an ascending trend since 2008, and as of 2020, consists of over 5.20 million kilometers of roads due to rapid urbanization and economic growth [18]. Asphalt is a thermoplastic material that demonstrates viscoelastic properties under most pavement operative conditions, thus playing an important role in pavement performance [19].

In an attempt to improve the durability and reliability of asphalt pavements to meet the climatic, traffic, and other requirements, the use of modified asphalt instead of raw asphalt has long been recommended as an effective approach [20]. It has been known for some time that virgin polymers can improve asphalt performance, especially of the high-temperature stability [21]. But virgin polymer materials are difficult to find and are uneconomical when used as modifiers [22]. High construction costs, when combined with awareness regarding environmental stewardship, have encouraged the use of waste plastics in asphalt modification.

Various studies and research projects have been conducted to find appropriate applications of using waste plastics in asphalt production, discussing the properties of waste plastic-modified asphalt, modified mechanisms, and environmental concerns [23,24]. In general, there is a desire to improve the utilization of waste plastic materials in asphalt, as long as performance is not adversely impacted [25]. According to evidence from previous literature, utilizing waste plastic as a modifier in asphalt production provides asphalt with a similar property to virgin polymers, substantially reducing the construction cost, and protecting the environment from additional contamination [26,27,28].

This paper is a literature review that critically presents the recent progress, developments, and challenges in the application of waste plastic-modified asphalt binder and mixture technologies. This paper is not the first review of this topic, but it renews the latest developments in the field. It focuses on the approaches for waste plastic-modified asphalt and mixture production, the influences of the main factors including the types and dosages of waste plastic, blending conditions and pretreatment methods on the properties of modified asphalt binder, and the discussion of the performance of waste plastic-modified asphalt mixtures, life cycle assessment (LCA) and practical engineering applications. In addition, compared with other reviews, this paper discusses the current challenge for waste plastic-modified asphalt including the stability, low-temperature performance, the modification mechanism, and laboratory problems of the asphalt blends, as well as provides potential ways to improve the properties of waste plastic asphalt and mixtures.

## 2. Waste Plastic and Sources

Plastics are synthetic materials derived primarily from refined crude oil petroleum products [25,29]. Table 2 provides a summary of common waste plastic products that can be recycled in accordance with ASTM D7611 (ASTM 2019) and GB/T 37547-2019. In general, the main sources of waste plastic in the environment are plastic containers, plastic packaging, and other common plastic industrial products, as shown in Table 2. Specifically, most single-use plastic products, such as bottles, packaging and disposable products, are manufactured from low-density polyethylene (LDPE) [30], high-density polyethylene (HDPE) [31,32], and polystyrene (PS) [33], while long-term plastic items are made from polyethylene terephthalate (PET) [28], polypropylene (PP) [34], polyvinyl chloride (PVC) [35,36], ethylene-vinyl acetate copolymer (EVA) [37,38] and others. Additionally, researchers have investigated other waste plastic types (e.g., polyurethane (PU) [39] and acrylonitrile butadiene styrene (ABS) [40,41]).

Different types of waste plastic have drastically different characteristics, which are mainly affected by chemical composition, chemical structure, and average molecular weight [26]. For example, the characteristics of LDPE are mainly affected by a large number of branched chains, and the crystallinity is only 55–65% [50]. By contrast, HDPE only has a few short branched chains, but the crystallinity is 80–90%—much higher than that in LDPE—so it is difficult to immerse in asphalt [51]. The melting point is suggested as one of the primary characteristics used to determine whether a waste plastic can be used as an asphalt modifier. There is an obvious difference among the melting points of these waste plastics, as listed in Table 2. PET has a melting point of around 260 °C, which is much higher than the temperatures for typical asphalt binder production and storage. PVC also does not meet the necessary criteria, as its melting point is 160–210 °C. If waste plastics with significantly different melting points are heated together, some will melt while others do not. Some plastics may not have melted yet, while others may be about to reach their decomposition temperature. Moreover, the performance of the mixed waste plastics may vary, with some plastics degrading when several types of plastic are heated together. Thus, it is necessary to classify and recover waste plastics before utilization. It is recommended that waste plastics such as PET, PVC, and PS are more suitable for dry process modification, because their melting points are higher than the heating temperature for preparing modified asphalt [42]. Waste plastics such as LDPE, HDPE, PP, and EVA, which have melting temperatures below the production temperatures of typical asphalt, are preferred for use in wet processes as modifiers (or potential modifiers) in asphalt production [52].

## 3. The Use of Waste Plastics in Asphalt

### 3.1. Forms of Waste Plastics Used in Asphalt

**Figure 2:**
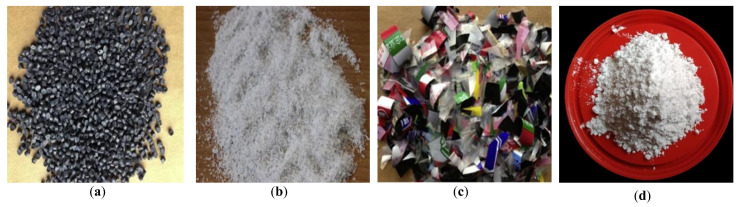
Various forms of waste plastics used as modifiers: (**a**) pellet [57]; (**b**) shredding [54]; (**c**) flake [57]; (**d**) powder [58]. Reprinted with permission from Refs. [54,57,58]. Copyright 2014 Elsiver publisher.

Waste plastics can be used as asphalt modifiers in a variety of forms through further processes. In the early days, waste plastics were processed into pellet form (Figure 2a) and were intended to be incorporated directly into the asphalt production plant [29,53]. These pellets were produced from 100% waste plastics, with sizes measuring between 0.3 mm and 0.5 mm [9]. In recent years, waste plastics have begun to be processed into shredding form, as shown in Figure 2b. However, the waste plastics in both pellet and shredding forms can only be processed through the complicated industrial system. Researchers have recently produced waste plastics as a modifier in more accepted forms by using common methods such as scissors and crushers in the lab. For example, Modarres and Hamedi [54] cut waste PET bottles and cans into small pieces (larger than shredding) and crushed them into flake form using a special crusher, as shown in Figure 2c. Kumar and Garg [55] and Lin et al. [56] made modified asphalt with waste plastics in thin strip form (20 × 3 mm^2^) and fiber form (less than 2 mm). Furthermore, waste HDPE powder and electronic-plastic (e-waste) powder (as shown in Figure 2d) have been used as asphalt modifiers [32,40].

### 3.2. Approaches to Incorporating Waste Plastics into Asphalt

There are two main approaches used to incorporate waste plastics into the asphalt: the wet process and the dry process [9]. In the wet process, waste plastics are added directly into the asphalt binder at high temperatures, where mechanical mixing is required to achieve a homogeneous plastic-modified binder blend. The mixing temperature and mixing time depend on the nature of the waste plastic source and asphalt binder. In the dry process, waste plastics are added directly to the asphalt mixture, either as a partial aggregate replacement or a mixture modifier [59]. When the addition of waste plastic is carried out by a wet process, the waste plastics are added to the asphalt binder to modify their properties before coming into contact with the aggregates [60]. When the plastics are added using a dry process, the waste plastics are mixed with aggregates so that they actually act as reinforcement materials [9].

Both the wet and dry methods have advantages as well as drawbacks, as shown in Table 3. The wet process is a conventional way of adding waste plastics, whereby they are mixed with the asphalt in a high-shear mill. Thus, the wet process requires specialized mixing and storage facilities [61], and it is better for controlling the properties of the modified asphalt binder [62]. This is likely the reason why the wet method is currently the most widely used in asphalt modification. By contrast, the dry process does not require professional equipment. It can be applied in any asphalt plant without major modifications [9]. The results from previous research have shown that the modified asphalt binder produced by the wet process has a higher viscosity, which allows a better coating of the aggregate particles, without exudation or drainage problems [63]. The modified asphalt mixture produced by the dry method has relatively poor water stability [62]. In terms of cost, the AC-16 mixture production materials using the dry method costs around CNY 290,000 per kilometer, which is lower than the wet method [64].

The performances of asphalt mixtures containing different waste plastics are significantly different because of the wet or dry process. Overall, the asphalt mixtures containing waste HDPE and EVA show similar properties for both the wet and dry processes. However, the waste HDPE mixture produced using the dry process exhibits poor water sensitivity [62].

Most previous studies focused on the wet process. The wet process is currently the most widely used for polymer asphalt modification because of its enhanced thermal behavior. However, the dry process is more cost-effective and has a simpler production process, meaning it is more convenient for waste plastic-modified asphalt production. Thus, further research is needed due to the lack of normative guidance and engineering experience for the dry process.

### 3.3. Single and Composite Modification

#### 3.3.1. Single Modification

Each type of waste plastic has its own chemical composition, unique structure, molecular weight, etc., all of which affect the performance of modified asphalt [26] when such plastics are used as a modifier independently in asphalt production.

(1) Waste LDPE

LDPE has a lower specific gravity, strength, and temperature resistance than HDPE because of its long, flexible, and linear polyethylene chain [65]. Due to the irregular structure of the multimolecular chain arrangement of LDPE, the branched chains in asphalt combine with each other to form reticular three-dimensional structures, which can better improve the properties of modified asphalt. Thus, LDPE is widely used as a modified material for asphalt. Since the 1990s, several studies in China, Europe, the US, and the UK have reported the use of modifiers made by recycled LDPE independently [66,67]. Khan et al. [68] studied waste LDPE, HDPE, and crumb rubber (CR) as an addition to base bitumen, and showed that modified asphalt binder with 10% LDPE offers the best resistance against rutting compared to HDPE and CR. Ho et al. [69] investigated combinations of three types of recycled LDPE as asphalt modifiers. The results have shown that the molecular weight and molecular weight distribution of waste LDPE have significant effects on the asphalt’s low-temperature performance, thermal storage stability, and polymer phase distribution. The recycled LDPE with lower molecular weight and wider molecular weight distribution is more suitable for asphalt modification, compared with high molecular weight LDPE with very narrow molecular weight distribution.

(2) Waste HDPE

As discussed above, the high crystallinity of HDPE makes it difficult to immerse in asphalt, which also affects the compatibility of modified asphalt. It is agreed that the waste HDPE-modified asphalt has higher stiffness and viscosity, and better moisture resistance [70]. Costa et al. [37] indicated that waste HDPE-modified asphalt has higher stiffness and lower penetration, but worse resilience and creep recovery, compared with SBS-modified asphalt.

(3) Waste PP

Recycled PP-modified asphalt has the common characteristics of thermoplastic polyester modified asphalt, especially the superiority of high-temperature performance. However, the addition of waste PP reduces the ductility of modified asphalt and decreases the fatigue cracking performance [71]. Specifically, the reduction of the ductility is around 20% when 5% of waste PP is added to the asphalt [71,72]. Thus, it is recommended that waste PP-modified asphalt is suitable for high-temperature and high-humidity areas, but the viscosity needs to be improved [45].

(4) Waste PVC

Recent studies have shown that the addition of waste PVC increases the viscosity and stiffness of base asphalt so that the modified asphalt has better rutting resistance. One possible reason is that the chloride and carbon bond dipole in PVC provides a greater stiffness [73]. A study by Ziari [74] indicated that waste PVC improves the fatigue resistance, but the thermal cracking resistance is poor. It is noteworthy that hydrogen chloride (HCL) can be formed and discharged into the atmosphere when PVC is heated to a high temperature [26]. Thus, measures should be taken to avoid air pollution.

(5) Waste PET

According to the Wellness Recovery Action Program (WRAP), PET is one of the most recycled plastic wastes [47]. Because of the high melting point, most researchers tend to use waste PET for dry modification [75]. Results have shown that waste PET-modified mixtures developed using the dry method have an improved high-temperature performance and reduced fracture resistance when the dosage of PET is 30% and 50% [76].

(6) Waste PS

Waste PS-modified asphalt mixtures developed using the dry process are found to have higher rigidity, but this could be a problem in colder areas in terms of cracking resistance [33]. Specifically, waste PS exhibits the lowest elastic behavior in the modified asphalt mixture compared with waste PE, PP, and rubber asphalt mixture using the dry process [42]. Fang et al. [77] successfully used a very low-density PS waste to expand the stiffness of asphalt and improve its rutting resistance. Hasan et al. [71] indicated that the addition of waste high impact PS (HIPS) in asphalt improves the asphalt’s stiffness, but decreases the low-temperature properties. Furthermore, much more attention should be paid to the fact that harmful substances are released when PS is heated above 70 °C.

(7) Waste EVA

Waste EVA has good compatibility with asphalt, so it has been widely studied and applied. The results have shown that the large volume of the vinyl acetate group becomes a non-crystalline area or amorphous area, which plays a role similar to rubber when EVA is mixed with asphalt. The crystalline area of EVA has high stiffness, which acts as a reinforcing bar, and greatly improves the high-temperature stability, low-temperature cracking resistance, and viscosity of modified asphalt [78]. It also exhibits certain improvements in low-temperature performance when small amounts of waste EVA are added (2–4%) [79].

(8) Waste ABS

The most common e-plastics used in the manufacture of electronic devices are ABS. Evidence from recent studies indicated that the use of e-plastic powders for asphalt modification helped improve the asphalt’s viscosity and blending and mixing temperatures, meanwhile decreasing rutting susceptibility compared to virgin asphalt [80]. The low-temperature performance of ABS-modified asphalt is equivalent to that of virgin asphalt binders [81]. Compared with waste EVA- and PE-modified asphalt, waste ABS has poor performance as an asphalt modifier, but it seems to have better storage stability [26]. Moreover, the pavement performance of waste ABS-modified asphalt is better than unmodified asphalt [82]. According to the Mechanistic-Empirical Pavement Design Guide (M-E PDG) [83], using e-waste materials as modifiers for asphalt mixtures using the dry method would decrease the design thickness of the asphalt layers [84].

(9) Waste PU

Bazmara et al. [85] used thermoplastic PU and synthetic PU as modifier additives in asphalt production. The results showed that the addition of synthetic PU increased the asphalt’s viscosity and stiffness. Both types of PU improved the performance of base asphalt at high temperatures, including high rutting resistance and performance grade; however, they had no notable effects on asphalt performance at low temperatures. A similar result was reported by Cong [86], who noted that waste PU-modified asphalt had good deformation resistance, aging resistance, fatigue resistance, and high-temperature storage performance. Waste PU-modified asphalt mixture developed using the wet method also had excellent water stability and deformation resistance [87]. Hot-mix asphalts with PU-modified bitumen yielded improvements in stability and lower deformation [88]. With regard to the PU-modified mixture, Salas et al. showed that, compared with the virgin sample, the PU-modified MA from the wet method exhibited lower indentation, and thus the modified mastic asphalts (MA) can be used for heavy-traffic roads [89].

**Figure 3:**
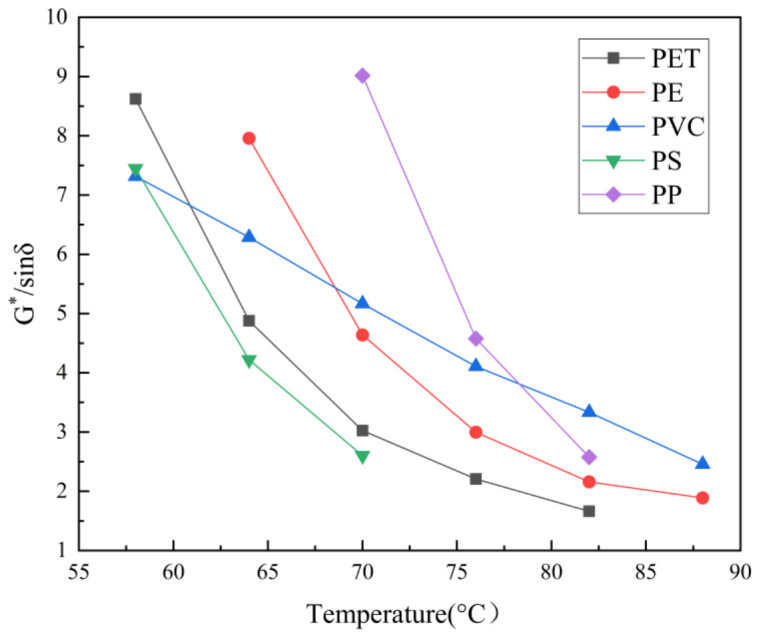
Rheological results of various waste plastic-modified asphalts [36,91,92,93,94].

Table 4 presents a summary of the effects of waste plastics on asphalt performance with respect to compatibility, high- and low-temperature performance, and viscosity, based on the most recent literature reviewed in this paper. It is evident that the addition of waste plastics can most likely increase the high-temperature stability and viscosity as well as decrease the low-temperature flexibility. Waste LDPE, PP, EVA, ABS, and PU have good compatibility with asphalt compared with other types of waste plastics, which can be seen from the summary of the rheological results of various waste plastic-modified asphalt in Figure 3. The high-temperature rheological property of PP-modified asphalt was the best, followed by PE- and PVC-modified asphalt, and PS-modified asphalt was the least effective. However, further research is needed due to the difference in dosage and asphalt.

#### 3.3.2. Composite Modification

The application of waste plastic as an independent modifier in asphalt is rare in current studies and engineering practices. This is because the key properties of asphalt cannot be improved by using only one type of waste plastic. In order to enhance and optimize the properties of waste plastic-modified asphalt binder to meet the needs of increased traffic demands, there has been growing interest in composite modification. Recently, more and more studies have investigated the properties of modified asphalt binders containing waste plastic and various materials [95]. This interesting trend means that the application of waste plastic as an asphalt modifier has been accepted by researchers and engineering practice. Some researchers investigated modified blends containing two or more types of waste plastic. For instance, Brovelli et al. [96] and García-Morales et al. [97] assessed the high-temperature stability of base asphalt modified by combining LDPE and EVA. Lai et al. [98] studied the compatibility and performance of waste HDPE/LDPE/PP-modified asphalt. Other researchers focused on the application of various modified blends of waste plastics and common polymers. Nasr and Pakshir [99] tested three melt-compounding combinations of waste PET and crumb rubber to improve the rutting and fatigue damage resistance of two base asphalt binders. A study reported by Al-Abdul Wahhab et al. [44] suggested that waste LDPE/HDPE-modified asphalt, in combination with an elastomeric SBS, can obtain higher recovery and strain resistance, which are better than using the same amount of SBS alone. Additionally, Krzysztof et al. [100] improved the conventional and thermal properties of asphalt by blending waste LDPE, ground tire rubber (GTR), and elastomer. Other studies have reported that waste plastics can be mixed with some common materials such as sulfur [101,102], carbon black [103], and polyphosphoric acid [104,105] as asphalt modifiers.

## 4. Factors Affecting Properties of Waste Plastic-Modified Asphalt

### 4.1. Waste Plastic Properties

The characteristics of waste plastics, such as type, chemical composition and structure, and molecular weight, affect the time required for blending, as plastics with higher molecular weight require more time to blend homogeneously with the asphalt binder. Additionally, waste plastics are produced in smaller sizes to help disperse and dissolve into the asphalt binder [94].

PE is one of the most popular thermos-plastics, and it is one of the earliest waste plastics to be used as an asphalt modifier in the world. PE has the simplest polymer structure, with each carbon atom connected to two hydrogen atoms. PE is categorized based on density into HDPE, LDPE, and Linear LDPE (LLDPE) [106]. Evidence from the literature shows that, compared with HDPE, the intermolecular force in LDPE is weaker, which is beneficial to the compatibility between asphalt and LDPE [31,32,50]. It is consequently suggested that both HDPE and LDPE are appropriate for asphalt modification, but LDPE is better.

In recent years, various types of waste plastics have been used as an asphalt modifier. Hu et al. [92] reported that the rheological properties of asphalt binder are enhanced when using waste packaging tape PP as the modifier. Gürü et al. [107] confirmed that thin liquid polyol PET (TLPP) and viscous polyol PET (VPP) made from waste PET bottles can improve the low-temperature performance and fatigue resistance of the modified asphalt. Furthermore, Köfteci et al. [108] found a significant difference in the performances of asphalt binders modified by different waste PVC sources (window, blinds, and cable wastes).

### 4.2. Asphalt Binder Properties

Asphalts are composed of two main phases: (1) an oily phase consisting of saturated hydrocarbons, aromatic cyclic products, and resins, and (2) a non-oily phase formed by asphaltenes and carbenes; however, the chemical composition and structure are different [109]. Elemental analyses indicate that most asphalts contain 79–88 weight percent (wt%) carbon, 7–13 wt% hydrogen, 2–8 wt% oxygen, traces to 8 wt% sulfur, and traces to 3 wt% nitrogen. Asphaltene contents provide a basis for the classification of asphalts into sol- or gel-types. In general, an asphalt low in asphaltene content (5–10 wt%) has properties characteristic of sol-type asphalt, and has high-temperature susceptibility, high ductility, and a low oxidative hardening rate. In contrast, asphalt with high asphaltene content (20–30 wt%) is gel-type, and has low-temperature susceptibility, low ductility, and a susceptibility to oxidative age hardening. Certainly, asphalt with intermediate asphaltene contents has properties intermediate between the sol- and gel-type behavior [110,111,112]. A study reported by Lesueur [113] shows that high asphaltene content decreases the compatibility between polymer and asphalt. Furthermore, Giavarini et al. [114] found that modified asphalt’s properties depend not only on the difference in density and viscosity between asphalt and polymer, but also on asphalt structure.

### 4.3. Waste Plastic Dosage

The utilization of suitable waste plastics in asphalt modification shows an improvement in asphalt properties; however, there is an optimum point between the waste dosage and asphalt properties. Many studies available investigated the effects of modified asphalt containing waste plastics at various dosages on its properties and pavement performance. The dosage range of common polymer-modified asphalt is between 2.5 wt% and 3.5 wt%, with a higher dose range greater than 7 wt% being referred to as highly modified asphalt [115]. Mashaan et al. [28] reported that the ideal content of waste plastic is 6–8 wt% to improve the rutting and aging resistances of modified asphalt. Naskar et al. [90] found that modified asphalt with 5 wt% waste plastic has the highest thermal stability compared to the other binders investigated. However, the penetration, softening point, and elasticity of the binder are negatively affected when the waste plastic content is up to 7 wt%. Fuentes-Audén et al. [116] also concluded that only low waste plastic concentrations (0–5 wt%) can be used for road paving whereas high waste plastic concentrations (10–15 wt%) are suitable for roofing. A similar study by Fernandes et al. [117] reported that increasing the waste plastic content in base asphalt improves the softening point temperature, resilience, and viscosity. Moreover, Karmakar and Roy [118] and Ameri et al. [79] indicated that an increased modifier content negatively affects the compatibility between modifier and asphalt, and low-temperature performance. Therefore, the authors recommended that a waste plastic concentration of approximately 5 wt% is better for asphalt properties [21]. Specifically, the optimal percentages of waste PET, PVC, and PP are 3.47 wt%, 6.25 wt%, and 4.64 wt%, respectively [95]. Briefly, the optimal dosage of waste plastics in modified asphalt is not only highly dependent on the properties of the waste plastic and base binder, but also the specific requirements of the asphalt binder.

### 4.4. Blending Conditions

The results from recent studies indicated that the blending conditions (blending temperature, blending time, and blending speed) used for asphalt modification strongly affect the asphalt properties [78,112,119]. Many studies have been conducted to find the optimum blending conditions for various waste plastic-modified asphalt production. García et al. [120] found that the stirring speed for asphalt preparation determines the size of the polymer particles and thus the rheological properties of modified asphalt. Babalghaith et al. [121] suggested that the optimal blending time for common modified asphalt to achieve the best rheological properties is about 30 min. Fang et al. [57] reported that the optimum parameters for waste PE-modified asphalt preparation were a shear rate of 3750 revolutions per minute (rpm), a temperature of 150 °C, and a shear time of 1.5 h. Another study found a longer blending time of 6 h [97].

The optimum blending conditions used for various types of waste plastic-modified asphalt are summarized in Table 5. The selection of mixing methods mostly depends on the specific type of waste plastic and its content use—the optimum content is about 3–6 wt%. For these seven types of waste plastics, the blending temperature is about 150 to 180 °C, blending time is between 1 and 3 h, and blending speed is from 1200 to 5000 rpm.

### 4.5. Pretreatment Methods for Waste Plastic

It is necessary to carefully consider the appropriate pretreatment method for waste plastic before it is added into the asphalt, as it affects the compatibility between the asphalt and modifier. Generally, pretreatment methods can be divided into two main methods: the physical method and the chemical method. It seems that most researchers (approximately 85%) prefer using the physical method to pretreat waste plastics, according to all of the related research papers referenced in this review.

#### 4.5.1. Physical Method

Waste plastics can be processed into smaller particles through physical methods such as grinding, shredding, pulverization, and extrusion.

Table 6 shows a comparison of the common physical methods used for pretreating waste plastics. Waste plastics can be pretreated using a suitable physical method into various forms of smaller size, based on their sources and purpose of use. For example, waste PET bottles can be processed into particles of larger or smaller sizes by crushing or grinding.

#### 4.5.2. Chemical Method

The use of the chemical method for the preparation of waste plastic is commonly carried out through grafting or irradiation to provide better compatibility and properties with the modifier, so that the performance of modified asphalt is improved. Li et al. [125] successfully increased the reaction of LDPE and asphalt through grafting LDPE with glycidyl methacrylate (GMA) containing carbon double bonds, epoxy groups, and epoxy functional groups, resulting in improved performance of the modified asphalt. Vargas et al. [43] improved the asphalt performance at a higher temperature using a similar method of grafting HDPE with glycidyl methacrylate. Yeh et al. [126] reported a similar result by grafting maleated PP.

In recent studies, a novel method named irradiation was used for pretreating waste plastic. Ahmedzade et al. [127] found that the chemical interaction between the waste HDPE and asphalt could be caused by irradiated waste HDPE, consequently enhancing the physical properties of the asphalt. Thus, the irradiated HDPE used as a modifier is recommended. A similar result was achieved by replacing electron beams with gamma rays [128].

## 5. Engineering Properties, Environmental Concerns and Practical Engineering Applications of Waste Plastic Asphalt Mixtures

### 5.1. Engineering Properties

Most studies have demonstrated that the addition of waste plastics significantly increases the stiffness and rutting resistance of base asphalt and mixture, and thus, has the potential to extend the service lives of asphalt pavements [31,68,99,129]. Asphalt mixtures modified with waste PE, PP, and rubber have a similar performance of increased rutting resistance [130]. Specifically, the rut depth values are reduced by more than half for waste EVA-modified asphalt mixtures, and by up to a third for waste HDPE-modified asphalt mixtures compared with conventional asphalt mixtures [62]. The performance of waste HDPE-modified mixture in terms of fatigue resistance is similar to or slightly worse than that of the conventional mixture, while the performance of the waste EVA mixture shows significant improvement. Both of the waste PE- and PP-modified mixtures have superior durability and properties with higher resistance to permanent deformation and moisture damage [131]. The waste PVC mixture shows a stronger rutting resistance by improving the rheological properties of the asphalt binder [132].

Based on the results of laboratory tests on asphalt binders, it can be expected that highly modified asphalt mixtures will have better functional properties, durability, and anti-rutting resistance [133]. Laboratory results indicate that asphalt mixtures containing 20% PP/rubber have better low- and high-temperature performances and water sensitivity than those with SBS modified asphalt mixtures [134].

Currently, the main types of waste plastic-modified asphalt mixture are stone matrix asphalt (SMA) and asphalt concrete (AC). Plastic gives an increase in stability, split tensile strength, and compressive strength compared to the conventional SMA mixture [135]. But the waste plastic might increase the cracking potential of the mixture [136]. Laboratory tests indicated that SMA mixtures containing waste plastic increased resistance, anti-rutting performance, fatigue resistance [137], and moisture damage [138]. However, the effect of PET on the moisture susceptibility of SMA mixtures was found to be negligible [139]. Some researchers studied the performance of AC mixtures by adding waste plastic and found that the mixture exhibited an increase in rutting resistance [130,140,141] and Marshall stability and stiffness [130,140,141], and a reduction in the moisture damage resistance, workability, fatigue resistance [141,142], and thermal susceptibility [140].

Some scholars have tried to use waste plastics in the reclaimed asphalt pavement (RAP) mixture, which also gives us an alternative way to alleviate the environmental problems caused by plastics. The results have shown that the addition of waste plastic materials into the RAP mixture potentially improves the durability of the pavement [143]. By contrast, the study of Yamin [144] showed that the addition of RAP has a negative effect on durability compared with the conventional mixture. The result of Leng’s [145] research indicated that the samples containing RAP- and PET-derived additives offered better overall performance than conventional mixtures, increasing the rutting resistance by at least 15% and fatigue cracking resistance by more than 60%. Therefore, the rutting resistance and fatigue cracking resistance performance of the RAP mixture with waste plastics have been improved, and the durability remains to be further studied.

### 5.2. Environmental Concerns

The environmental benefit is obvious, even if the total amount of waste plastic is a small part of the total asphalt mixture. Studies have shown that the CO_2_ equivalent emissions can be decreased by 10.2% when 8% of virgin PP is replaced with the same amount of waste PP, and the emission decreases by 15.6% when waste PP is used to replace SBS. More evidence is needed to show that the implementation of these waste plastics into the pavement is eco-friendly [146].

The LCA method is typically applied to quantify the environmental impacts of using waste plastics in asphalt and asphalt mixtures throughout their entire life cycle—from raw material extraction, to transport, manufacturing, and use. Poulikakos et al. [147] assessed four hypothetical roads using the LCA method, with considerable savings in cost, CO_2_, and energy compared to conventional asphalt mixtures using all of the virgin components. Another study showed that the energy consumption can be reduced by 2.23% when an asphalt mixture with waste plastic instead of a conventional asphalt mixture is used as a surface course material [148]. Santos [149] also obtained similar conclusions by using waste PP instead of virgin PP. Bart evaluated the environmental behavior of asphalt mixtures with waste PET using the LCA method. The results showed that the use of waste PET in asphalt pavements not only benefits in terms of energy saving and reduced greenhouse gas emissions, but also improves the resistance of cracks, thereby decreasing maintenance requirements [147]. The cradle-to-gate LCA modeling and sensitive analysis suggest that highly modified asphalt mixtures (containing 20% PP/rubber) are more eco-friendly in terms of energy consumption and greenhouse gas emissions [134].

Multi-attribute analysis methods, including environmental factors, costs, and engineering properties, have been conducted to investigate the overall problem of plastic recycling on the road for mixture sustainable factors [149]. The research combined the laboratory experimental performance with the environmental LCA results using the multi-attribute grey relational analysis (GRA) method, and comprehensively sorted the scheme, providing an innovative perspective for the study of recycled materials for road and pavement engineering. Yu et al. [134] evaluated the waste PP asphalt mixture and SBS asphalt mixture from environmental concerns, using cradle-to-gate LCA modeling. The results indicate that the waste PP asphalt mixture is more eco-friendly compared with the SBS asphalt mixture. Two highly modified asphalt mixtures, replacing 25% of bitumen with two types of plastic waste, show environmental and economic advantages; specifically, a 17% reduction in environmental impact and an 11% reduction in economic impact can be achieved [150].

### 5.3. Practical Engineering Applications

Some researchers have taken a step in evaluating the performance of waste plastic-modified asphalt mixtures by assessing actual field performance, not only at the laboratory level.

(1) India

Waste plastics have been widely used in road construction in India. Since 2002, more than 2500 km of asphalt concrete pavements have been modified with polymer waste using the dry method. In the following years, six sites were selected to investigate the status of waste plastic asphalt pavement. The results showed that these roads worked well, without potholes, raveling, and rutting [151].

The Indian Highway Congress published guidance for the use of waste plastics in the bituminous mixture in 2013, while the National Rural Highway Development Agency provided guidelines on the use of waste plastics in rural road construction [151].

(2) UK

MacRebur’s recycled waste plastic was incorporated into the asphalt instead of traditional bitumen and used by Durham County Council in the UK for resurfacing a section of A689 near Sedgefield, and for resurfacing runways and taxiways at Carlisle Airport in the UK.

The Department for Transport provided £1.6 million to extend Cumbria’s existing road that is built from recycled plastic mixed with asphalt. The trial will also produce a guidance document on the use of plastic asphalt.

(3) USA

UC San Diego built the first asphalt road made with recycled plastic binder as opposed to a petroleum-based bitumen binder in the USA.

(4) Africa

The local government is building the first plastic road in the city of Kouga municipality, South Africa. It is reported that a 1-kilometer ‘plastic road’ could consume nearly 700,000 plastic bottles or 1.8 million plastic bags.

(5) The Netherlands

In June 2021, the PlasticRoad Company built the first parking spaces and residential street using waste plastic in the municipality of Almere; in the same year, waste plastic was used to construct a car-sharing location in the province of Overijssel and the municipality of Hardenberg, and to build a bike path at Delft University of Technology.

(6) Mexico

The modular design of Mexico City’s PlasticRoad offers temporary water storage and drainage features. 

The use of waste plastics on actual roads has just started, and most plastic roads are piloted in bicycle lanes, runways, and rural roads. Only the waste plastic roads in Africa have follow-up reports, and the long-term outcomes of many pilot roads are not yet clear.

## 6. Challenges of Using Waste Plastics with Asphalt

### 6.1. Low-Temperature Performance

There is no doubt that the utilization of waste plastics with asphalt significantly improves the asphalt’s high-temperature performance [9,31,129]. However, it increases the creep stiffness of asphalt at low temperatures, resulting in the degradation of the low temperature cracking resistance of asphalt at the same time [50]. Rubber is an elastomer and can help improve the low-temperature crack and fatigue resistance of asphalt. As a consequence, the invention of a modifier prepared by mixing waste plastic and rubber has been proven successful to solve this problem.

Studies reported by Yan et al. [152] and Kalantar et al. [21] show a great improvement of both the high- and low-temperature performances of asphalt modified with waste PE and waste rubber blending. The researchers explained this phenomenon through laboratory experiments. Ma et al. [153] found a significant enhancement of the compatibility of asphalt modified with waste plastic/rubber blends compared with adding plastic and rubber independently or with additional compatibilizer. Other researchers give an explanation that the elastic component of rubber powder compensates for the side effects of plastic due to its continuous elastic network formed by the rubber/plastic composite modified asphalt [154].

However, the evaluation index of waste plastic-modified asphalt on low-temperature performance is controversial. The assessment of asphalt’s low-temperature performance in China is mostly based on the Bending Beam Rheometer test and the ductility test. Yang [50] suggested that the use of ductility, as an index for evaluating the asphalt’s low-temperature performance, has a certain limitation in the evaluation of modified asphalt. The results from Yu [155] lead to a similar conclusion: the network structure of plastic-modified asphalt samples prepared and stored at low temperatures can reduce the crack extension and improve the toughness of asphalt, which is contradictory to the result of the ductility test. This means that the ductility is not enough to evaluate the low-temperature performance of asphalt. Further studies should be performed to develop an assessment system for the performance of waste plastic-modified asphalt.

### 6.2. Storage Stability

Poor storage stability is one of the main drawbacks of polymer-modified asphalt [156]. It is particularly prominent in asphalt with composite modification. The compatibility between modifier and asphalt binder is one factor that strongly influences the storage stability of a modified asphalt binder [153].

Composite modified asphalt is a multiphase system. The composite modifier is an intimate blend that comprises at least two incompatible polymers forming a two- or multi-phase blend including waste plastic and other polymers. Phase separation could occur caused by the asymmetric molecular dynamics among the components in the composite modifier [157]. The modifier and asphalt binder are also incompatible due to the great differences in the density, structure, and molecular weight, from the point of view of thermodynamics [158]. Only when the compatibility among the polymers and asphalt is high enough to avoid phase separation in the binder can a proper, good quality pavement be achieved [21]. Experimental results confirmed that the addition of sulfur, carbon black, hydrophobic clay minerals, nano clay, and montmorillonite along with waste plastic into a modifier can significantly improve the compatibility of asphalt. Functionalization is another method to solve the compatibility problem. As shown in Table 7, functionalization refers to the addition of specific functional groups, free radicals, or grafting in the polymer to make chemical reactions occur in the blend to improve the specific functions of asphalt. For example, grafting LDPE with glycidyl methacrylate (GMA) and epoxy functional groups can successfully increase the reaction between LDPE and asphalt, thus improving the compatibility with the asphalt [125]. In addition, the preparation of waste LDPE and SBS blends by Gao et al. [2] improved the storage stability by forming a small amount of copolymer, which was similar to the effect of a compatibilizer obtained through a chemical reaction between the functional groups.

### 6.3. Microcosmic Modification Mechanism

**Figure 4:**
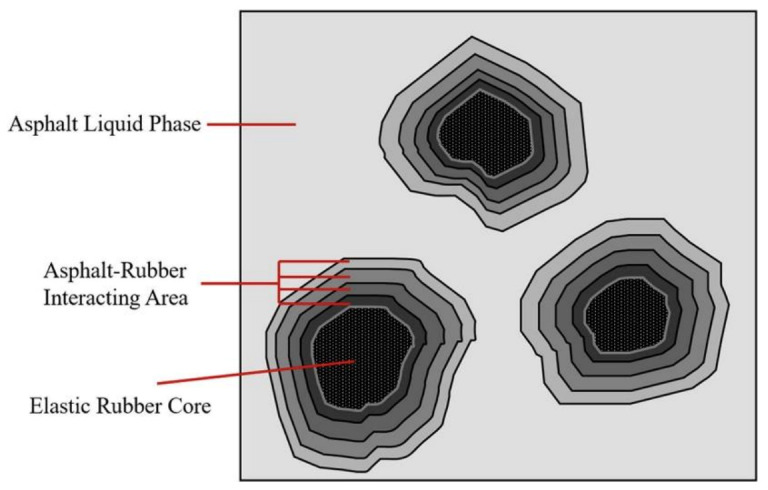
Swelling mechanism of rubber-modified asphalt [168]. Reprinted with permission from Ref. [168]. Copyright 2020 Elsevier publisher.

The modification mechanism of polymer on asphalt is complex. The interaction principle of the waste polymer has not been comprehensively and systematically investigated. Most scholars have successfully explained the modification mechanism of waste polymers and base asphalt by the “swelling mechanism”. As can be seen from Figure 4, the waste polymer absorbs the light components in the base asphalt, which subsequently swells and agglomerates together, forming a thick gel layer around it. This is the third phase interface between the polymer phase and the base asphalt phase, forming a partially compatible system, during the mixing process of the waste polymer and base asphalt [167]. It has been demonstrated that the swelling of the polymer is the key point for asphalt modification, and sufficient swelling ensures the formation of the interface layer [168]. The interface layer is a third phase between the two phases in terms of composition, structure, and performance, which makes the blend system stable and provides special performance [168].

Fang et al. [169] found that PVC absorbs the light components of asphalt, and swells in asphalt and forms the network structure, affecting the physical properties of the modified asphalt. A further conclusion is that the swelling of waste LDPE and the network structure of the blend helps improve the properties of the base asphalt [65]. Similarly, the swelling of waste PS and waste PE in asphalt is caused by the absorption of the low molecular composition of asphalt, forming a network structure, thereby improving the viscoelasticity and high-temperature performance of the asphalt [129].

Microscopic technology and infrared spectroscopy are effective tools for helping scholars to investigate the modification mechanism. The modifier and binder in the system may go through physical blending or chemical reaction, and the process is difficult to define. Fourier transform infrared spectroscopy (FTIR) can be used to qualitatively and quantitatively analyze the composition of functional groups of materials according to the principle that specific functional groups and chemical bonds selectively absorb infrared light at specific wavelengths, to clarify the modification mechanism of asphalt [170].

The use of FTIR can quantify the absorption bands. Some researchers found that there was no change in the main functional groups of waste PVC asphalt binder, indicating the modification is a physical effect [169]. Other researchers reported that there are slight increases in absorption peaks at 2925 cm^−1^ and 2856 cm^−1^ because the modified asphalt contains a large number of methyl and methylene groups, which coincide with the absorption peaks of the asphalt before modification. However, other functional groups do not change before and after modification, indicating that the waste LDPE modification of asphalt is also a physical process [46].

Microtechnologies, including scanning electron microscopy (SEM) [126,155], atomic force microscopy (AFM) [116,118], and confocal laser scanning microscopy (CLSM) [43,111], are also used to characterize the morphology of modifiers and modified asphalt. It is observed by CLSM that the morphology of the polymer/asphalt system moves from a continuous asphalt-rich phase, with a dispersion of polymer molecules, to a continuous asphalt-rich phase, with a dispersion of asphalt globules, with an increase in the amount of blended polymer [111].

**Figure 5:**
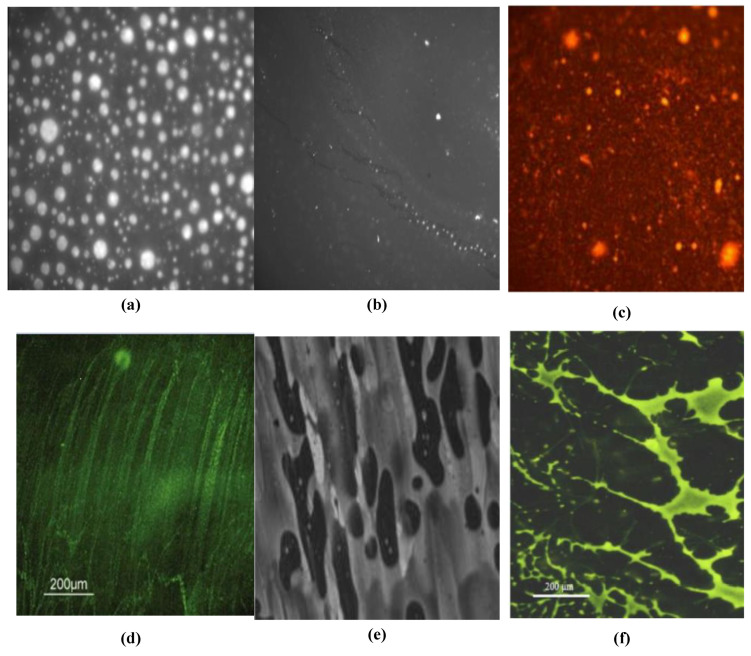
Fluorescence microscopic images of blends of asphalt with different types of waste plastics: (**a**) LDPE/asphalt [43]; (**b**) HDPE/asphalt [43]; (**c**) PP/asphalt [45]; (**d**) PVC/asphalt [46]; (**e**) EVA/asphalt [49]; (**f**) PS/asphalt [77]. Reprinted with permission from Refs. [43,45,46,49,77]. Copyright 2013 Elsevier publisher.

The dispersion of modifiers and the structure form can be seen clearly in the microscopic image. This shows the impacts of modifiers on asphalt and reveals the modification mechanism. This paper summarizes the fluorescence microscopic images of different types of plastics, as shown in Figure 5. The morphology of asphalt/LDPE is shown in Figure 5a. The LDPE phases are dispersed in the asphalt phase in the form of nearly spherical particles, and the edges of the particles become blurred by swelling. In the HDPE/asphalt blend, HDPE is less compatible with asphalt than LDPE, the HDPE phase is limited in extent, and there is no visible connection between the particles (Figure 5b). In the PP/asphalt blend, the yellow PP phases become the dominant phases covering most areas, and the brown asphalt (background) begins to fade and the boundary is blurry (Figure 5c). The morphology of PVC in asphalt is long stripe shape (Figure 5d), while the EVA and PS are branched network structures accompanied by some dispersed granulated particles, which is beneficial to improve the properties of asphalt (Figure 5e,f).

### 6.4. Laboratory Operational Problems

The uneven dispersion and aggregation of modifiers may be encountered in the laboratory during the modification process of waste plastics. Since asphalt binder is colloidal material, the agglomeration phenomenon naturally appears [171]. The dispersion and aggregation of modifiers in asphalt significantly affect the performance of waste plastic-modified asphalt and its further application [172].

The aggregation of modifiers can be improved by improving the mixing conditions to a certain extent. As described in Section 4.4, ‘The Influence of Blending Conditions’ in this review, the mixing conditions of different types of waste plastics are different, and the appropriate mixing time, temperature, and speed need to be determined. Similarly, changing the size of the modifier can improve the problem of modifier aggregation. Smaller particle sizes have a relatively larger surface area per unit mass of the modifier and can help the modifier to achieve a homogeneous mix, thus overcoming phase separation and improving the stability of the modified asphalt [173,174].

## 7. Conclusions

Waste plastic binders and mixtures are gaining increasing attention due to their engineering performance and economic and eco-friendly benefits. This paper presented a review of the waste plastics most commonly used in asphalt binders and mixtures, analyzed and compared various approaches for waste plastics-modified asphalt and mixture production, and discussed the influence of the main factors on the properties of modified asphalt and mixtures. The paper discussed the current challenges for waste plastic-modified asphalt, such as the stability, low-temperature performance, the modification mechanism, and laboratory problems. Based on this review, the following points can be drawn:

(1) The use of waste plastic as an asphalt modifier expands the application field of waste plastic and avoids the waste of resources. It is also an effective way to solve the waste plastic disposal problem and reduce environmental pollution. However, more attention should be paid to PS and PVC, as these plastics produce harmful emissions when heated at high temperatures.

(2) The source of waste plastics is one of the main factors that affect the performance of the modified asphalt due to the differences in chemical composition and structure, resulting in different basic characteristics. High melting point plastics, such as PVC, PS, and PVC, are more suitable for the dry processes; LDPE, HDPE, PP, and EVA, with low melting points, are more appropriate for the wet processes.

(3) Waste plastic can be made into various forms and sizes depending on the specific purpose when used for asphalt modification. Waste plastic with a smaller size and larger surface area, combined with composite modification, is recommended to achieve better high- and low-temperature performance and storage stability.

(4) A physical or a chemical modification can be accurately distinguished upon comparing the FTIR spectra before and after the asphalt modification. The microstructure of the modified asphalt, modification process, and mechanism can be further characterized using microcosmic technologies such as SEM, AFM, and CLSM. Previous studies have provided evidence that asphalt modified with waste plastic independently is a physical modification.

(5) The use of the LCA method can effectively quantify the environmental impacts of using waste plastics for asphalt and asphalt mixture modifications. Waste plastic-modified asphalt mixtures indeed reduce energy consumption and emissions, and the implementation of these waste plastics into the pavement is eco-friendly. The use of waste plastics in actual roads has just started, therefore the long-term outcomes require further assessment.

(6) There is no doubt that the high-temperature performance of waste plastic-modified asphalt is quite effective. However, there are still limitations for waste plastic-modified asphalt applications due to the separation between the waste plastic modifier and the asphalt, and the poor low-temperature performance of waste plastic-modified asphalt. Pretreatment methods (grinding, grafting, irradiating) and composite modification (additives, functionalization) can improve the disadvantages of waste plastic-modified asphalt. However, due to the cost and application limitations, waste plastic asphalt modification requires suitable pretreatment and modification methods.

## 8. Future Recommendations

As discussed above, waste plastic-modified asphalt is an important approach currently available to reduce environmental impact and natural resource depletion, and it deserves more attention to improve the interaction between the modifier and the asphalt to improve the asphalt properties and pavement performance.

(1) Most of the studies presented in this review focus on assessing the properties of waste plastic-modified asphalt, but the microcosmic mechanism of asphalt modification has not received enough attention. Several studies report results that are characterized by micro-experiments, but the microcosmic mechanism of waste plastic modification is rarely involved. The microcosmic mechanism of waste plastic-modified asphalt is still not well understood, and more attention should be paid to address this knowledge gap.

(2) There is a need to better understand and control the effects of using different types of waste plastic and other polymers as modifiers on the properties of the asphalt, and find an optimum proportion for the modifier. This will promote the application of recycled waste plastics in modified asphalt.

## Figures and Tables

**Table 1:** Laws and main measures in different countries or areas [17].

Countries or Areas	Laws and Main Measures
USA	In 2019, California Conference Bill No. 1080 phased out disposable plastics by 2030.
Canada	It was announced that the use of disposable plastic products would be banned from 2021.
Japan	Enactment of the ‘Plastic Resource Recycling Promotion Act’ in 2019 to reduce disposable plastic products by 25% by 2030.
Africa	South Africa introduced a plastic bag levy policy in 2003; plastic shopping bags are banned in 16 countries in West Africa.
India	From 2 October, 2019, the use of plastic bags, bottles, plates, and straws was prohibited nationwide.
UK	The ban on the use of plastic straws, plastic cotton swabs, etc., began on 1 October 2020.
Europe	The European Commission’s proposal to prohibit the use of disposable plastic products, covering ten kinds of disposable plastic products, was enacted on 3 July 2021 with a view to reducing disposable plastic containers and packaging in Europe by 2030.
Norway	From 3 July 2021, the use of disposable plastic products such as plastic straws and tableware is prohibited.
Iceland	From 3 July 2021, it is prohibited to put commonly used disposable plastic products on the market.
China	In 2007, the General Office of the State Council of China published a Notice for limiting the use of free plastic bags. In 2020, the National Development and Reform Commission and the Ministry of Ecological Environment of China published a Notice for a ban on the use of disposable plastic products.

**Table 2:** Melting point and main sources of waste plastics.

Type	Melting Point (°C)	Sources [21,42]
LDPE	110–120 [43]	Soft drink and mineral water bottles
HDPE	130 [44]	Plastic bottles and packaging
PP	145–165 [45]	Straw, furniture, and wrapping industries
PVC	160–210 [46]	Fittings and plumbing pipes
PET	260 [47]	Soft drink and water bottles
PS	210–249 [48]	Disposable plates and cups, carry-out containers, and compact disc cases
EVA	65–80 [49]	Soles, thin films, and wire cables
ABS	No true melting point [42]	Electronic devices
PU	No true melting point [42]	Upholstered furniture and mattresses, shoes, cars, medical devices, buildings, and technical equipment

**Table 3:** The advantages and drawbacks of different processes.

Method	Production Cost	Technological Problem		Performance of Mixture	
		Advantage	Drawback	Advantage	Drawback
Wet process	Expensive (AC-16)	Normative guidance and engineering experience	Complex production process (specialized mixing and storage facilities)	Higher viscosity	Poor storage stability
Dry process	Cheap (AC-16)	Lack of normative guidance	Simple production process (no need of professional facility)	-	Poor water stability

**Table 4:** Characteristics of common waste plastics.

Type	Characteristics of Waste Plastic				Reference
	Compatibility	High-Temperature Stability	Low-Temperature Flexibility	Viscosity	
LDPE	√	√	-	√	[30]
HDPE		√	-		[31,32]
PP	√	√	-	√	[34,45,90]
PVC	-	√	-	√	[35,36]
PET	-	√	-	√	[28]
PS	-	√	-	√	[33]
EVA	√	√	√	√	[32,37,38,78]
ABS	√	√	-	-	[26]
PU	√	-	-	√	[85,87]

**Table 5:** Blending conditions commonly used for waste plastic-modified asphalt.

Waste Plastic	Optimum Content (wt.%)	Blending Temperature (°C)	Blending Time (min)	Blending Speed (rpm)	Reference
PE	3–6	145–190	60–150	1750–4000	[31,32,106,122]
PP	3–6	160–180	45–90	1800–4000	[45,72,90,123]
PVC	4–8	160–180	60–180	1300–2000	[36,108]
PET	2–8	180	60	13,000	[37,38,56,78]
EVA	3–5	140–180	80–120	1800–3000	[37,38,78]
PS	4–6	150–190	90–120	3000	[33,77]

**Table 6:** Comparison of physical methods for pretreating waste plastics.

Type	Source	Physical Method	Form	Size	Reference
Waste plastic bag waste plastic pipe	-	Shredding	Strip Fiber	1–2 cm 20 × 3 mm^2^	[35,55,90]
Waste plastic bottle	PET	Cutting and crushing	Particle	0.45–1.18 mm	[54]
Waste milk bag	-	Extruding	Pellet	-	[57]
Waste plastic bottle waste express bag	PET	Grinding	Particle Piece	0.45–0.5 mm 2–5 mm	[56,124]
Waste window blind and cable	PVC	Pulverization	Powder	-	[108]

**Table 7:** The influences of additives and functionalization on the improvement of compatibility.

Method		Modifying Influence	Reference
Additive	Phosphoric acid	Improve rheological behaviors of modified asphalt and increase the storage stability at the storage temperature.	[159]
	Montmorillonite	Improve the storage stability of modified asphalt and does not compromise its excellent high temperature rheological properties.	[155]
	Nano clay	Improves the stability of modified asphalt.	[160]
	Hydrophobic clay minerals	The storage stability of modified asphalt is improved by reducing the density difference between polymer modifiers and asphalt.	[161]
	Carbon black	Reduces the density difference between polymer and asphalt, thus improving the storage stability of modified asphalt.	[103,162]
	Sulfur	In this process, the loss of unsaturation, the shift of the double bonds and a molecular isomerization occur.	[101,102,163]
Functionalization	Copolymer	Improves the compatibility of modified asphalt.	[2,164,165]
	Cross-linking agent	Makes the polymer react with asphalt, so as to provide a chemical connection between the two and form a three-dimensional network structure and improve the compatibility of asphalt.	[38,93]
	Radical initiator	Promotes direct covalent molecular bonding between e-waste plastic powders and the modified asphalt.	[41]
	Antioxidants	Believed to play a role by scavenging free radicals and decomposing the hydroperoxides generated during oxidation.	[166]
	Functional groups	It is generally expected that the added functional groups will interact with some components of asphalt in various ways, such as forming hydrogen bonds or chemical bonds, which may improve compatibility to some extent.	[24]
	Grafting	Maleic anhydride (MAH), methacrylic acid (MAA) and glycidyl methacrylate (GMA) were used to graft some currently used polymer modifiers and were found to improve the storage stability of asphalt.	[24]

## Data Availability

Not applicable.
